# Supplementary material for: HIVprotI: an integrated web based platform for prediction and design of HIV proteins inhibitors
Source: J Cheminform. 2018 Mar 9;10:12. doi: 10.1186/s13321-018-0266-y (PMC5845081; doi:10.1186/s13321-018-0266-y)
Supplement: Supplementary file 2 — Additional file 2. Source code of HIVProtI web server. [file 13321_2018_266_MOESM2_ESM.zip › HIVprotI_Source-code/hivprotI/weka-3-6-6/doc/weka/gui/graphvisualizer/package-summary.html]

weka.gui.graphvisualizer


---


|  |  |  |
| --- | --- | --- |
| **Overview** \| **Package** \| Class \| **Tree** \| **Deprecated** \| **Index** \| **Help** \| **Weka's home** | |  |
| **PREV PACKAGE**   **NEXT PACKAGE** | **FRAMES**    **NO FRAMES**     **All Classes** |  |


---

## Package weka.gui.graphvisualizer

| **Interface Summary** | |
| --- | --- |
| **GraphConstants** | GraphConstants.java |
| **LayoutCompleteEventListener** | This interface should be implemented by any class which needs to receive LayoutCompleteEvents from the LayoutEngine. |
| **LayoutEngine** | This interface class has been added to facilitate the addition of other layout engines to this package. |

| **Class Summary** | |
| --- | --- |
| **BIFParser** | This class parses an inputstream or a string in XMLBIF ver. |
| **DotParser** | This class parses input in DOT format, and builds the datastructures that are passed to it. |
| **GraphEdge** | This class represents an edge in the graph |
| **GraphNode** | This class represents a node in the Graph. |
| **GraphVisualizer** | This class displays the graph we want to visualize. |
| **HierarchicalBCEngine** | This class lays out the vertices of a graph in a hierarchy of vertical levels, with a number of nodes in each level. |
| **LayoutCompleteEvent** | This is an event which is fired by a LayoutEngine once a LayoutEngine finishes laying out the graph, so that the Visualizer can repaint the screen to show the changes. |
| **Messages** | Messages. |

| **Exception Summary** | |
| --- | --- |
| **BIFFormatException** | This is the Exception thrown by BIFParser, if there was an error in parsing the XMLBIF string or input stream. |

---


|  |  |  |
| --- | --- | --- |
| **Overview** \| **Package** \| Class \| **Tree** \| **Deprecated** \| **Index** \| **Help** \| **Weka's home** | |  |
| **PREV PACKAGE**   **NEXT PACKAGE** | **FRAMES**    **NO FRAMES**     **All Classes** |  |


---
